# Nanoparticle Albumin-Bound-Paclitaxel in the Treatment of Metastatic Urethral Adenocarcinoma: The Significance of Molecular Profiling and Targeted Therapy

**DOI:** 10.1155/2014/489686

**Published:** 2014-08-18

**Authors:** Yasmin M. Abaza, Carlos Alemany

**Affiliations:** ^1^Department of Internal Medicine, Florida Hospital, Orlando, FL 32804, USA; ^2^Cancer Institute of Florida, Florida Hospital, 2501 North Orange Avenue, Suite 689, Orlando, FL 32804, USA

## Abstract

Primary urethral cancer is rare and accounts for only 0.003% of all malignancies arising from the female genitourinary tract. Due to the rarity of this disease, no consensus exists regarding the optimal therapeutic approach. Nanoparticle albumin-bound-paclitaxel has been shown to be effective in the treatment of a number of malignancies including metastatic breast, pancreatic, and bladder cancer. We present a 67-year-old woman with advanced metastatic urethral adenocarcinoma resistant to two lines of chemotherapy (ifosfamide/paclitaxel/cisplatin and irinotecan/5-fluorouracil/leucovorin) that showed a dramatic response to nanoparticle albumin-bound-paclitaxel. This is the first case report to document the use and efficacy of nanoparticle albumin-bound-paclitaxel in the treatment of unresectable metastatic urethral cancer.

## 1. Introduction

Primary urethral cancer is rare and accounts for approximately 0.02% of all female cancers [1]. The most common histological type of urethral cancer is transitional cell carcinoma (TCC) followed by squamous cell carcinoma (SCC) and adenocarcinoma [2]. About 98% of women are symptomatic at presentation complaining of obstructive or irritative voiding symptoms, hematuria, dyspareunia, and a palpable urethral mass [3]. Since the clinical presentation is nonspecific, diagnosis is often delayed until advanced stages of the disease. Due to the rarity of the disease, no consensus exists regarding the optimal therapeutic approach. Nanoparticle albumin-bound (nab) paclitaxel has been shown to be effective in the treatment of metastatic breast, pancreatic, and bladder cancer [4–6]. We report a case of metastatic urethral adenocarcinoma that showed a significant response to nab-paclitaxel.

## 2. Case Report

A 67-year-old Hispanic woman diagnosed with advanced metastatic adenocarcinoma of the urethra presented to our institute for a second opinion. Her condition started with symptoms of frequency, urgency, urinary incontinence, and pelvic discomfort. She was initially treated for urge incontinence but subsequently developed acute urine retention prompting further investigation. Physical examination revealed a firm submucosal periurethral mass measuring 3 cm in diameter inseparable from the anterior vaginal wall. Abdominal and pelvic CT scan and MRI showed a circumferential periurethral mass with evidence of metastasis to the pelvis, peritoneum, liver, bone, and pelvic lymph nodes. Cystoscopy failed to show any intraluminal pathology. CT-guided biopsy revealed moderately differentiated adenocarcinoma of the urethra. She was started on 5 cycles of ifosfamide, paclitaxel, and cisplatin (ITP regimen). CT scan and MRI preformed after therapy showed mild decrease in the size of the metastases suggestive of partial response to therapy. Nevertheless, after 6 months her disease progressed and she was referred to hospice care, which the patient declined.

She presented to our institute for a second opinion. Tissue biopsy was obtained and sent for molecular profiling to help direct therapy. Molecular profiling displayed overexpression of SPARC, TOPO1, c-kit, and PDGFRB and under expression of TS, ERCC1, and GART. Nab-paclitaxel was thought to be a good therapeutic option given the presence of SPARC overexpression which increases the concentration of paclitaxel in the tumor interstitium leading to selective tumor cell apoptosis.

However, due to insurance issues, the patient was initiated on modified IFL chemotherapy which consists of irinotecan (125 mg/m^2^), 5-fluorouracil (500 mg/m^2^), and leucovorin (20 mg/m^2^). After 8 cycles, her disease progressed and weekly nab-paclitaxel (100 mg/m^2^) was initiated. This dose was based on the results of two studies conducted on patients with metastatic breast cancer. A phase II study performed by Blum et al. compared 100 mg/m^2^ to 125 mg/m^2^ weekly nab-paclitaxel in patients with metastatic breast cancer that have been heavily pretreated with taxanes. This study showed that weekly administration of 100 mg/m^2^ nab-paclitaxel was as effective as 125 mg/m^2^ and had a more favorable side effect profile [7]. Another randomized phase III study conducted by Seidman et al. found that weekly administration of paclitaxel was more effective than triweekly in patients with metastatic breast cancer [8]. Given that our patient was heavily pretreated with taxanes we found this regimen to be the most suitable for her condition.

**Figure 1:**
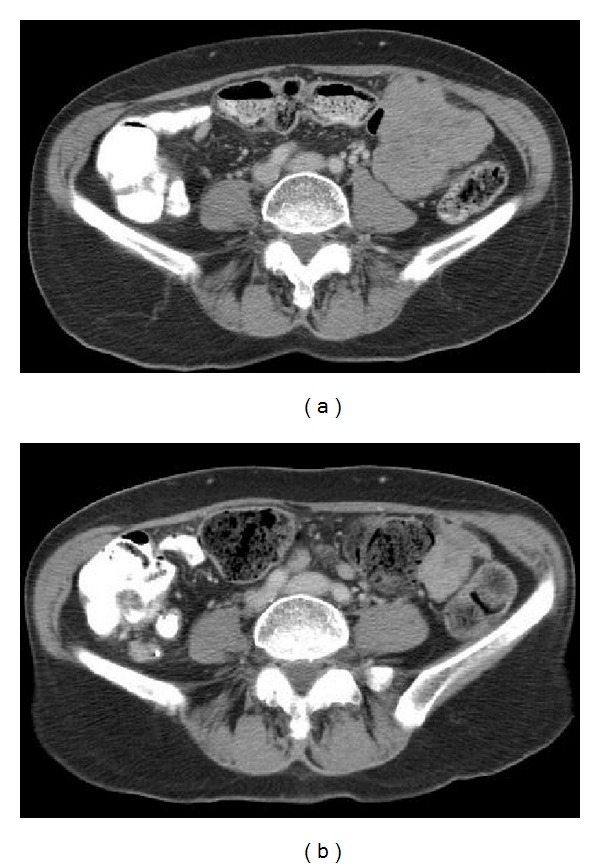
The figure shows the dramatic response of metastatic urethral cancer to nanoparticle albumin-bound (nab) paclitaxel. CT scan performed before therapy (a) showed an 11.5 cm metastatic mass in the left pelvis. This mass decreased to 3.5 cm after 5 months of nab-paclitaxel (b).

Nab-paclitaxel was well tolerated except for the development of grade 1 peripheral neuropathy and chemotherapy-induced anemia. Dramatic improvement was noted after 5 months of therapy with 70% reduction in the size of the pelvic masses on CT scan (Figure 1). Nab-paclitaxel was continued until disease progression providing about 19 months of progression free survival. Ultimately, nab-paclitaxel was discontinued and pemetrexed (500 mg/m^2^) introduced.

## 3. Discussion

Primary urethral cancer is three times more common in males than females, with a reported incidence rate of 4.3 per million and 1.5 per million, respectively [2]. Diagnosing urethral cancer can be a challenge requiring thorough physical examination under anesthesia, cystourethroscopy, and either CT scan or MRI of the abdomen and pelvis [3]. Biopsy of suspicious lesions is essential for establishing the diagnosis and determining the histological subtype. Prognosis is poor and depends largely upon the stage and location of the tumor. Unlike tumors of the proximal urethral, tumors of the distal urethra have better outcomes given their greater accessibility for resection and earlier presentation [3].

Literature available on primary urethral cancer is scarce and based mainly on small case series and reports. Given its rarity, histological heterogeneity, and advanced stage at presentation, no consensus exists regarding the optimal systemic therapy. The chemotherapeutic regimen used is based mainly upon the underlying histological subtype. Cisplatin and 5-fluorouracil are the most commonly used regimens for SCC [3, 9]. TCC is treated like metastatic urothelial cancer using gemcitabine and cisplatin [10]. However, there is controversy regarding the optimal regimen for advanced urethral adenocarcinoma. A retrospective study conducted by Dayyani et al. reported efficacy of cisplatin-based chemotherapeutic regimens for patients with advanced urethral cancer, including SCC and adenocarcinoma [11]. The lack of optimal systemic chemotherapy for advanced urethral adenocarcinoma warrants the need to explore different novel agents in an attempt to improve outcomes.

Nab-paclitaxel, a Cremophor-free albumin-bound 130 nm particle form of paclitaxel, was approved by the Food and Drug Administration in 2005 for the treatment of metastatic breast cancer. Numerous studies have showed its efficacy in the treatment of metastatic breast, pancreatic, and bladder cancer [4–6]. The effectiveness of nab-paclitaxel is thought to be due to SPARC-albumin interaction. SPARC, a 43 kDa secreted protein, is a key regulator for numerous cellular functions including cell proliferation, survival, and cell migration. It is secreted by both cancer and stromal cells and is therefore highly expressed in the tumor-stromal interface of invading tumors. It has a high affinity for albumin and is induced by both hypoxia and acidity. Many tumors accumulate albumin for de novo protein synthesis. Being albumin-bound, nab-paclitaxel uses the gp60 and caveolae-mediated albumin transport pathway to traverse the endothelial lining of blood vessels entering the tumor interstitium where it is trapped by SPARC. As a result, the concentration of paclitaxel near tumor cells increases thereby inducing their selective apoptosis. Although SPARC overexpression is associated with tumor progression and poor prognosis, targeted therapy using nab-paclitaxel may improve outcomes in these patients [12].

In our patient, molecular profiling showed SPARC overexpression and, hence, the dramatic response to nab-paclitaxel. This underscores the significance of molecular studies in predicting prognosis and response to treatment, thereby guiding the selection of chemotherapeutic regimens. This is the first case report to document the use of nab-paclitaxel in the treatment of unresectable metastatic urethral cancer. Further studies are needed to assess the efficacy of nab-paclitaxel as first or second-line treatment for patients with advanced urethral cancer.

## Abbreviations

TCC: Transitional cell carcinoma
SCC: Squamous cell carcinoma
Nab-paclitaxel: Nanoparticle albumin-bound-paclitaxel
SPARC: Secreted protein, acidic, cysteine-rich
TOPO1: Topoisomerase inhibitor 1
PDGFRB: Platelet-derived growth factor receptor, beta polypeptide
TS: Thymidylate synthase
ERCC1: Excision repair cross-complementation group 1
GART: Glycinamide ribonucleotide transformylase
CT: Computed tomography
MRI: Magnetic resonance imaging.
